# Metabolome and Lipidome Profiles of *Populus* × *canescens* Twig Tissues During Annual Growth Show Phospholipid-Linked Storage and Mobilization of C, N, and S

**DOI:** 10.3389/fpls.2018.01292

**Published:** 2018-09-05

**Authors:** Mutsumi Watanabe, Florian Netzer, Takayuki Tohge, Isabel Orf, Yariv Brotman, David Dubbert, Alisdair R. Fernie, Heinz Rennenberg, Rainer Hoefgen, Cornelia Herschbach

**Affiliations:** ^1^Max Planck Institute of Molecular Plant Physiology, Wissenschaftspark Potsdam-Golm, Potsdam, Germany; ^2^NARA Institute of Science and Technology, Ikoma, Japan; ^3^Chair of Tree Physiology, Institute of Forest Sciences, Albert Ludwigs University of Freiburg, Freiburg, Germany; ^4^Chair of Ecosystem Physiology, Institute of Forest Sciences, Albert Ludwigs University of Freiburg, Freiburg, Germany; ^5^Department of Life Sciences, Ben-Gurion University of the Negev, Beersheba, Israel

**Keywords:** annual growth cycle, nutrient mobilization, nutrient storage, phospholipids, *Populus* × *canescens*

## Abstract

The temperate climax tree species *Fagus sylvatica* and the floodplain tree species *Populus* × *canescens* possess contrasting phosphorus (P) nutrition strategies. While *F. sylvatica* has been documented to display P storage and mobilization (Netzer et al., 2017), this was not observed for *Populus* × *canescens* (Netzer et al., 2018b). Nevertheless, changes in the abundance of organic bound P in gray poplar trees indicated adaptation of the P nutrition to different needs during annual growth. The present study aimed at characterizing seasonal changes in metabolite and lipid abundances in gray poplar and uncovering differences in metabolite requirement due to specific needs depending on the season. Seasonal variations in the abundance of (i) sugar-Ps and phospholipids, (ii) amino acids, (iii) sulfur compounds, and (iv) carbon metabolites were expected. It was hypothesized that seasonal changes in metabolite levels relate to N, S, and C storage and mobilization. Changes in organic metabolites binding P_i_ (P_org_) are supposed to support these processes. Variation in triacylglycerols, in sugar-phosphates, in metabolites of the TCA cycle and in the amino acid abundance of poplar twig buds, leaves, bark, and wood were found to be linked to changes in metabolite abundances as well as to C, N, and S storage and mobilization processes. The observed changes support the view of a lack of any P storage in poplar. Yet, during dormancy, contents of phospholipids in twig bark and wood were highest probably due to frost-hardening and to its function in extra-plastidic membranes such as amyloplasts, oleosomes, and protein bodies. Consistent with this assumption, in spring sugar-Ps increased when phospholipids declined and poplar plants entering the vegetative growth period and, hence, metabolic activity increases. These results indicate that poplar trees adopt a policy of P nutrition without P storage and mobilization that is different from their N- and S-nutrition strategies.

## Introduction

The annual growth cycle of temperate woody perennials is characterized by sequential periods of dormancy and active growth (Shim et al., 2014). A detailed view of the poplar annual growth cycle has been presented by Jansson and Douglas (2007) and Rohde and Bhalerao (2007). Dormancy release is followed by flowering, bud flush, and leaf expansion in spring. Wood formation starts in early summer concomitant with the development of leaves. In contrast to beech, poplar is a continuously growing tree species, which develops leaves throughout the entire vegetation period. After growth cessation in late summer, buds, which break in the following spring, start to develop. In autumn, leaf senescence precedes frost-hardening and ultimately dormancy. The factors and parameters controlling these periods and their correct succession are manifold. Day length perception via the phytochrome system (Coleman et al., 1991; Shim et al., 2014), temperature control of gene expression (Sjödin et al., 2008; Rennenberg et al., 2010; Wingler, 2015), endogenous control by chromatin remodeling, DNA and histone modification via methylation and acetylation (Karlberg et al., 2010; Conde et al., 2013; Shim et al., 2014), modulation of transcription factors (Ko et al., 2011) and phytohormones (Rohde and Bhalerao, 2007; Karlberg et al., 2010; Wingler, 2015) have been described to control the switch from dormancy to active growth and *vice versa*.

The annual growth cycle of deciduous trees further requires adaptation processes of whole tree nutrition in order to support the nutrient demand in spring (Millard and Grelet, 2010; Rennenberg and Schmidt, 2010). Nutritional demands are satisfied through an integrated system of internal nutrient storage, mobilization and transport, and root uptake. Nutrient resorption from senescing leaves with subsequent nutrient storage and mobilization is a special feature of deciduous trees and is considered an adaptation to nutrient limitation that allows fast development of new tissues in spring independent from current nutrient availability and uptake (Millard and Grelet, 2010; Rennenberg and Schmidt, 2010). During spring, nutrient uptake can be limited by low soil temperature, restricted microbial activity, and/or by competition with microbes and fungi in the rhizosphere (Gessler et al., 1998a; Simon et al., 2011, 2017). Nutrients resorbed from senescing leaves are stored as carbohydrates and storage lipids (Sauter and Kloth, 1987; Sauter et al., 1988; Sauter and van Cleve, 1989, 1994; Sauter and Wellenkamp, 1998), proteins and amino acids for nitrogen (Coleman et al., 1991; Gessler et al., 1998b; Cooke and Weih, 2005; Millard and Grelet, 2010; Wildhagen et al., 2010), sulfate and glutathione (GSH) for sulfur (Herschbach and Rennenberg, 1996; Dürr et al., 2010; Herschbach et al., 2012; Malcheska et al., 2013), and phosphate (P_i_) as well as organic-bound P_i_ (P_org_) for phosphorus (Netzer et al., 2017, 2018a). Broad leaf deciduous tree species store their nutrients in living cells of the twig, i.e., bark parenchyma as well as wood ray and pith cells (Sauter and Kloth, 1987; Sauter and van Cleve, 1994; Sauter et al., 1996; Sauter and Wellenkamp, 1998; Netzer et al., 2018b). Beside nutrient storage in the bark and wood of the twig, nutrients also accumulate in leaf buds established at the end of the vegetation period (Herschbach and Rennenberg, 1996; Netzer et al., 2017) and in seeds during their development (Hills, 2004; Borek et al., 2015). Nutrient resorption from senescing leaves and nutrient storage in twig tissues does not only prevent nutrient loss from the tree by leaf abscission (Rennenberg and Schmidt, 2010) and, hence, improves its nutrient use efficiency (Yuan and Chen, 2015; Netzer et al., 2017), but also allows conservation of nutrients for the whole ecosystem (Brant and Chen, 2015).

Phosphorus cycling during annual growth including storage and mobilization depends on the tree species, soil-phosphorus (P) availability, the growth habitat, i.e., the ecological niche, and on tree age (Netzer et al., 2017, 2018b, Plassard, 2018, Zavišić and Polle, 2018). Adult beech trees showed P storage and P remobilization in the twig as inorganic P_i_ and as P_org_ including phospholipids and glucosamine-6-phosphate (GlcN6P) in the bark and as N-acetyl-D-glucosamine-6-phosphate (GlcNAc6) in the wood (Netzer et al., 2017, 2018a). Leaf buds accumulate P_org_ as phospholipids at dormancy to support the P demand of developing buds in spring (Netzer et al., 2017, 2018a). In contrast, beech offspring at forest sites with low soil-P availability did not exhibit any P storage in stem bark and wood during dormancy, when P accumulated in leaf buds (Netzer et al., 2017). However, in a mesocosm experiment, beech offspring from a P-poor forest site clearly showed accumulation of P_org_ in the stem, while P_i_ accumulated in coarse roots of beech offspring from a P-rich forest (BBR) (Zavišić and Polle, 2018). Different to adult beech trees, adult poplar trees grown on P-rich soil did not exhibit P storage in the twigs, neither in bark or wood nor in the buds (Netzer et al., 2018b). Apparently, P_i_ uptake in spring seems to be sufficient to fulfill the P requirement of swelling and developing leaf buds. This finding is in clear contrast to N and S nutrition and seasonal S and N cycling previously observed for the same but juvenile poplar plants (Dürr et al., 2010; Wildhagen et al., 2010; Malcheska et al., 2013). S and N is stored as sulfate and in proteins during dormancy in the twig bark and wood and remobilized during spring to support swelling and bursting buds with N and S for growth and development. The absence of any P storage in poplar was attributed to (i) the fast growth of poplar, (ii) the P dynamics of the natural habitat of poplar, i.e., floodplains, (iii) the continuous growth pattern of poplar, and (iv) the adaptation to high soil-P_i_ availability (Netzer et al., 2018b; Plassard, 2018).

Although P storage does not seem to constitute a considerable feature in poplar, remodeling of organic-P compounds, particularly of phospholipids cannot be excluded due to different physiological demands during annual growth. The contribution of P_org_ to the total P (P_tot_) fraction in poplar differs between the seasons and among organs/tissues (Netzer et al., 2018b). During bud break when the percentage of P_i_ on P_tot_ amounts to only 20% in buds/leaves, P_org_ showed highest abundance and consequently constitutes the dominating P fraction at this time of the year. In addition, the P_org_ fraction showed highest levels during spring in the bark and was lowest during summer, when the P_i_ fraction was dominating (70% of P_tot_, Netzer et al., 2018b). These results show that the composition of P_org_ changes with specific patterns in a distinct organ/tissue in a season dependent manner. It can thus be hypothesized that changes in the P metabolite profile originate from the differences in utilization and demand during annual growth.

Spatiotemporal changes in metabolite abundance during leaf senescence, including lipid composition and contents, have been investigated in *Arabidopsis* leaves during seed filling (Watanabe et al., 2013). The decline in chloroplast lipids such as monogalactosyldiacylglycerols (MGDGs), digalactosyldiacylglycerols (DGDGs), and phosphatidylglycerols (PGs) has been identified as a senescence marker. A variety of studies investigated transcriptional changes and/or changes in metabolite abundances and proteome differences in poplar (Andersson et al., 2004; Keskitalo et al., 2005; Ruttink et al., 2007; Sjödin et al., 2008; Fracheboud et al., 2009; Karlberg et al., 2010; Ko et al., 2011; Larisch et al., 2012; Falasca et al., 2014). In addition, a recent review impressively summarizes the current knowledge in the release of dormancy for fruit trees buds (Beauvieux et al., 2018). All these studies investigated seasonal changes in one tissue only, i.e., bark, cambium, wood, buds/ leaves or berries, compared only two seasons, or compared tissues during a given season, respectively. Despite the knowledge from these investigations, seasonal changes over a whole annual growth cycle including spring, summer, autumn, and winter together with all twig tissues, i.e., buds/leaves, bark, and wood were described for beech trees, showing P storage and mobilization (Netzer et al., 2018a), but have not been reported for poplar that fulfill their P requirements by P uptake (Netzer et al., 2018b). Strong interactions between nitrogen and phosphorus nutrition in poplar (Gan et al., 2015) and of N and P resorption efficiency from senescent leaves in general (Han et al., 2013; Sohrt et al., 2018) have been described and seem to depend on carbon (C) metabolism (Sardans et al., 2012). Apparently, stoichiometric foliar N and/or P levels are strongly correlated to seasonal changes of the entire metabolome (Rivas-Ubach et al., 2012). Hence, the present study aimed at (i) identifying the P metabolites contributing to the seasonal changes in P_org_ of poplar twig organs/tissues and (ii) uncovering changes and interactions in C, N, and S storage together with the P metabolism during annual growth. It is hypothesized that poplar twigs, which do not reveal storage of P, show seasonal variation in sugar-Ps and phospholipid abundances that reflect differences in seasonal P metabolite requirements. It is further hypothesized that C, N, and S storage and mobilization is evident from changes in C metabolite, amino acid and sulfur compound abundances. Therefore, C, N, S, and P related metabolome and lipidome profiles were determined in poplar twig bud/leaves, bark, and wood in dormancy, spring, summer, and autumn.

## Materials and Methods

### Plant Material

The samples analyzed in the present study were the same samples as used in the work of Netzer et al. (2018b). Buds/leaves, bark, and wood from poplar twigs were obtained from a 7 years old (at the beginning of the present study) gray poplar (*Populus*×*canescens*) plantation close to the Chair of Tree Physiology (now Chair of Ecosystem Physiology) in Freiburg, Germany (coordinates 48.01470°N; 7.83252°E, 243.6 m a.s.l.) (Dürr et al., 2010; Wildhagen et al., 2010). In April 2006, poplar cuttings of the INRA clone 717 1B4 (Institute National de la Recherche Agronomique, INRA, France) were planted into the garden with artificial soil near the institute and were fertilized once with 120 g of a long-term fertilizer (Basacote Plus 12 M, COMPO, Austria) (Dürr et al., 2010). Leaves (or buds in winter), bark, and wood samples were obtained from twigs approx. 30 cm in length harvested from the sun exposed crown of five trees per harvest at approx. 10 m height using a pole saw in 2013/2014 as described in Netzer et al. (2018b). Sampling was carried out during two consecutive years with higher frequencies during spring and leaf senescence (Netzer et al., 2018b). Metabolome and lipidome analyses were performed with samples of buds/leaves, bark, and wood from 25 September, 2013 representing early autumn, 22 January, 2014 representing dormancy, 27 March, 2014 and 16 April, 2014 representing spring prior and after bud break, respectively, as well as 24 July, 2014 representing summer. Bud break was on the 1 April, 2014. Sampling was conducted at 9:30 am to prevent effects of diurnal changes. For the collection of leaf material, the first five mature leaves (counted from the top) were harvested and the petiole was removed from the lamina and discarded. Buds were harvested when present from the entire twig. Bark and wood samples were taken from the same section of the twig as leaves and were separated with a razor blade. Leaf, bark, and wood samples were rinsed with double distilled H_2_O (*dd*H_2_O) and dried with paper tissues to avoid contaminations. All samples were shock frozen in liquid nitrogen, homogenized to a fine powder under liquid nitrogen, and stored at −80°C until further analyses.

### Determination of Total C and N

Three mg of dried, fine milled and homogenized plant material was weighed into tin capsules using a precision scale. The samples were combusted in an elemental analyzer (vario PYRO cube, Elementar, Langenselbold, Germany) and analyzed in a continuous-flow isotope ratio mass spectrometer (isoprime precisION, Elementar, Langenselbold, Germany). The samples were measured against the reference standard IAEA-600 caffeine and IAEA-NO-3 potassium nitrate (repeated measurement precision was 0.10) for nitrogen, and IAEA-600 caffeine and IAEA-CH-3 cellulose (repeated measurement precision was 0.12) for C.

### Metabolite Extraction and Measurements

Metabolite extraction and analyses were performed as described previously (Watanabe et al., 2018). Approximately 50 mg of finely ground frozen material was homogenized in 300 μL of 100% methanol and then 200 μL of 100% chloroform was added. The polar fraction was prepared by liquid partitioning into 400 μL of ultra-pure water. After centrifugation, aliquots of 100 μL of polar phase and 40 μL of lipid phase were vacuum-dried. For measurement of ions, the dried polar phase was re-suspended in 550 μL of ultra-pure water. Ions were measured by an ICS-3000 system (Dionex, Idstein, Germany) with a KOH gradient for anion analysis and with a methanesulfonic acid gradient for cation analysis. For measurement of thiols and amino acids, the dried polar phase was re-suspended in 60 μL of 0.1 M HCl. Thiols and amino acids were measured by a combination of monobromobimane or *O*-phthalaldehyde fluorescent labeling, respectively, followed by high-performance liquid chromatography (HPLC) (UltiMate 3000, Dionex) with 20 μL of 0.1 M HCl extract. For measurement of primary metabolites by gas chromatography/time of flight-mass spectrometry (GC/TOF-MS), the dried polar phase was derivatized by methoxyamination and subsequent trimethylsilylation. Samples were analyzed using a Agilent 6890N gas chromatography system equipped with a VF-5ms capillary column (Agilent, Boeblingen, Germany) coupled to a Pegasus III TOF mass spectrometer (LECO Instrumente GmbH, Moenchengladbach, Germany). Peak area of the mass fragments was normalized on the basis of fresh weight of the sample. For measurement of chlorophylls, the dried lipid phase was re-suspended in 200 μL of ethanol. Chlorophyll content was determined using a spectrophotometric method to detect light absorbance reading at 665, 649, and 750 nm. For measurement of protein contents, the insoluble residue from the extraction was washed by 100% chloroform and then 100% methanol. The washed residue was re-suspended in 400 μL of 0.1 M NaOH and solubilized by heating at 95°C for 30 min. Protein content was determined using the dye-binding assay (Bradford, 1976) with bovine serum albumin (BSA) as reference.

For measurement of lipids by liquid chromatography/electrospray ionization-mass spectrometry (LC/ESI-MS), approximately 30 mg of finely ground frozen material was homogenized in 1 mL of a cold 1:3 (v/v) methanol:methyl-*tert*-butyl-ether solution that was subjected to constant shaking for 10 min at 4°C and sonication for 10 min using a ultrasonic bath with ice. Subsequently, phase separation was performed by adding 500 μL of a 1:3 (v/v) methanol:ultra-pure water solution and 500 μL of the upper lipid phase were vacuum-dried. The dried lipid phase was re-suspended in a 7:3 (v/v) acetonitrile:isopropanol solution. Samples were analyzed using a waters acquity ultra performance liquid chromatography (UPLC) system equipped with a C8 reverse-phase column (Waters, Eschborn, Germany) coupled to an Exactive Orbitrap (Thermo Fisher Scientific, Dreieich, Germany). Peak heights of the mass fragments were normalized based on the fresh weight of the sample.

## Results

### Variation of Nutrient Levels Are Suggestive of N and C, but Not P, Storage

**FIGURE 1:**
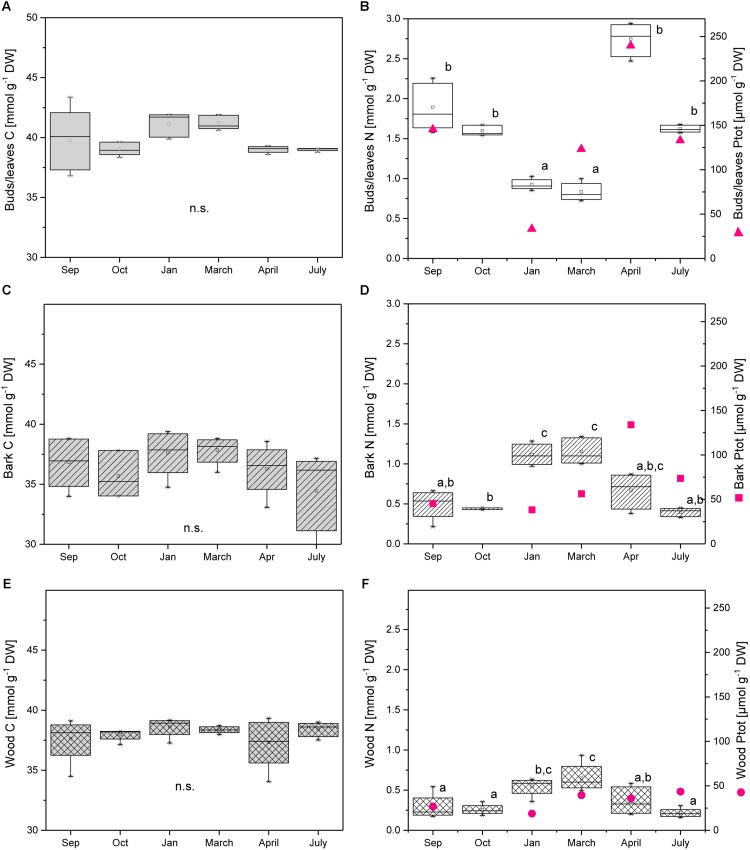
Total C, N, and P in buds/leaves, bark, and wood of adult poplar trees. *Box plots* of total C **(A,C,E)**, total N **(B,D,E)**, and total P (*magenta dots*, **B,D,F**) in poplar buds/leaves **(A,B)**, twig bark **(C,D)**, and wood **(E,F)**. Data of total P were taken from Netzer et al. (2018b). *Lower case letters* represent significant differences between seasons determined by one way ANOVA followed by the *post hoc* tests Bonferroni and Tukey’s (*P* < 0.05) per tissue (Sep = 09/26/2013; Oct = 10/02/2013; Jan = 01/22/2014; March = 03/27/2014; April = 04/16/2014; and July = 07/24/2014).

The C content in dry plant material was 45–50% in buds/leaves, similar in twig bark and wood (43–48%), and did not vary during annual growth (**Figure 1**). The C level in dormant buds as well as in buds during spring ranged from 39.8 to 41.9 mmol g^−1^ DW and was higher compared to young leaves in April after bud break (38.9 to 39.3 mmol g^−1^ DW) and in July (38.7 to 39.1 mmol g^−1^ DW). This result is in clear contrast to the N level that was lowest in buds/leaves during dormancy (0.85 to 1.03 mmol g^−1^ DW) and in early spring (0.72 to 1.0 mmol g^−1^ DW) and highest in late spring after bud break (2.47 to 2.94 mmol g^−1^ DW) (**Figure 1B**). A similar pattern was observed for the P_tot_ contents (**Figure 1B**; Netzer et al., 2018b). In twig bark and wood, the C level was similar throughout the annual growth cycle, whereas the N level was highest during dormancy and in early spring before bud break (**Figures 1C–F**). The clear and significant increase in N during dormancy indicates N storage during this time of the year, which was not found for P (**Figures 1D,F**; Netzer et al., 2018b). Total P was low during dormancy in the bark and similar to summer levels in the wood suggesting the absence of P storage.

### Characteristics of the Metabolome Profiles

**FIGURE 2:**
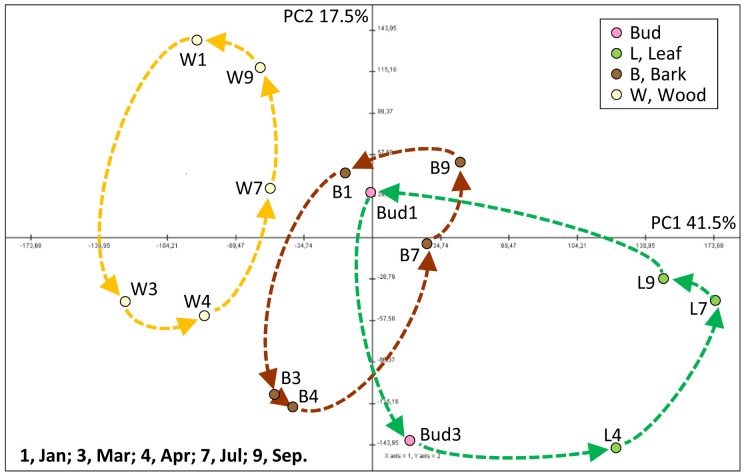
Principal component analysis (PCA) score plot of metabolite levels in buds/leaves, twig bark, and wood of adult poplar trees during annual growth. The plots were applied for 85 annotated metabolites including primary metabolites detected by GC/TOF-MS, ions by ion chromatography, amino acids and thiols by HPLC, and soluble protein by a Bradford method. PCA was conducted by the Multi Experiment Viewer (Saeed et al., 2003). PC, principal component. Data represent mean values of five biological replicates for each tissue at each time point.

Metabolome data raised from bud/leaf, bark, and wood organ/tissues were characterized by principal component analyses (PCA) asking whether the data sets provide specific differences between buds/leaves, bark, and wood as well as between seasons. The data sets separate into distinct units, which can be assigned to functional groups, when the data were normalized over all data (**Figure 2**). Principal component (PC) 1 explained 41.5% and PC 2 17.5% of the variance. PC1 separated wood, leaves and bud, and bark from one another. Remarkably, dormant buds in January and March match to the bark in January, July, and September, while green leaves were clearly separated from the bark and buds (**Figure 2**). PC2 separated the tissue samples by time indicating that metabolic compositions are distinct within a given tissue between the different harvest time points. Furthermore, each tissue showed a clear “cycle” when connecting the data points from January to March, April, July, and finally September, i.e., from dormancy (January) throughout the growth phase back to dormancy. These patterned structures resemble the physiological states and specificities of the investigated tissues.

**FIGURE 3:**
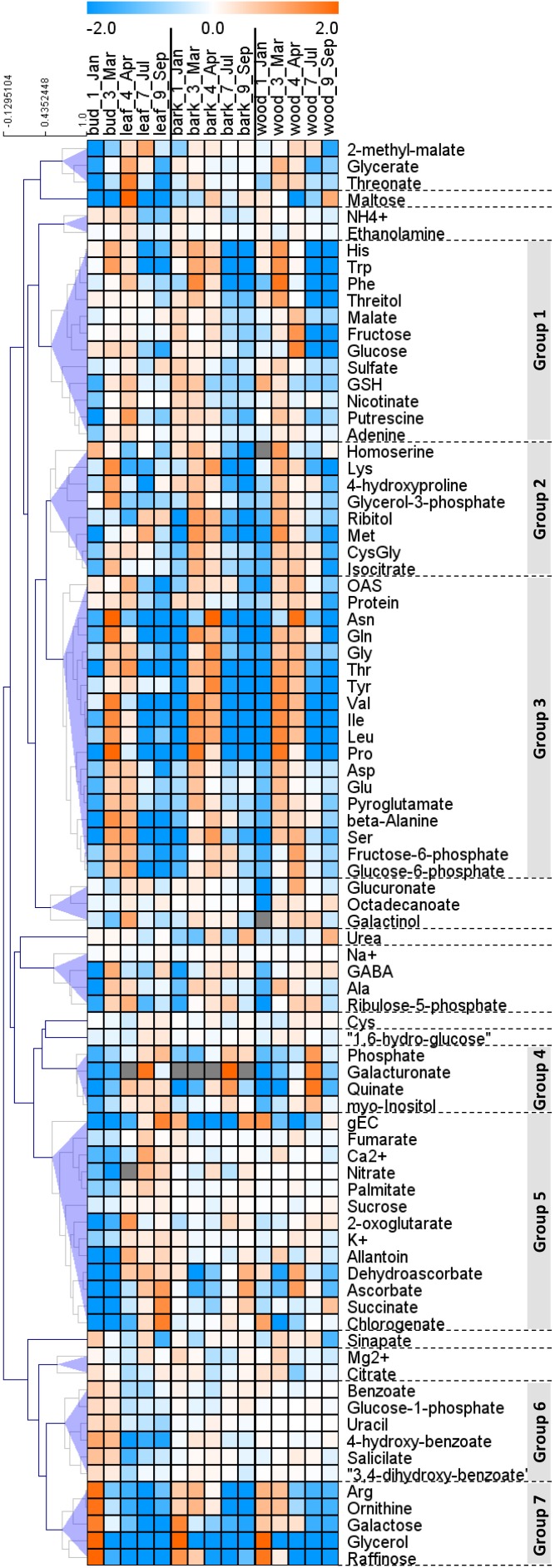
Hierarchical clustering (HCA) of metabolite changes in buds/leaves, twig bark, and wood of poplar during annual growth. Hierarchical clustering of the changes of 85 annotated metabolites. The 85 outer nodes were reduced to 17 by imposing a distance threshold, 0.5 (*shaded blue*). The groups highlighted in *gray* are described further in Section “Results.” Log2 ratios of fold changes in each tissue from the average values of all the seasons in each tissue are clustered using Pearson correlation by the Multi Experiment Viewer (Saeed et al., 2003). Data represent mean values of five biological replicates in each organ/tissue for each season. OAS, *O*-acetylserine; gEC, gamma-glutamylcysteine; GSH, glutathione; GABA, gamma aminobutyric acid.

In order to provide insight into the respective organ/tissue responses and to compare between buds/leaves, stem bark and wood, metabolome data were plotted as heat map with hierarchical clustering using Pearson correlation. Hierarchical cluster analyses provided seven groups that showed comparable responses, denoted group 1 to group 7 (**Figure 3**). Groups 1, 2, and 3 are dominated by amino acids that were highest during spring in all organs, i.e., buds/leaves, bark, and wood. Remarkable members are His, Trp, and Phe in group 1, homoserine, Lys, Met, and CysGly in group 2 and OAS, Asn, Gln, Gly, Thr, Tyr, Val, Ile, Leu, Pro, Asp, Glu, and Ser in group 3. Groups 4 and 5 were characterized by lowest levels in leaf buds in January and March before bud break and increasing abundance during the vegetation period after bud break. In contrast, bark and wood showed mixed responses without clear patterns. Members worth mentioning are the ions P_i_, nitrate, Ca^2+^, K^+^, and the metabolites galacturonate, myo-inositol, fumarate, 2-oxoglutarate (α-ketoglutarate), succinate, dehydroascorbate (DHA), ascorbate, chlorogenate. Group 6 showed seasonal changes only in leaves with highest levels in buds during dormancy and March. The bark and wood, in contrast, showed uniform abundances during the seasons. This group includes defense compounds benzoates but also glucose-1P. Metabolites of the group 7 showed highest levels in dormant buds and, in addition, high levels in the bark and wood during dormancy and in spring, all with strong characteristic decreases over the vegetative growth period toward July and September. Members are the N-rich compounds Arg and ornithine, NH_4_^+^ whose pattern is similar although changes were less pronounced, as well as galactose, glycerol, and raffinose. The following description aims at specifying these seasonal characteristics.

### Seasonal Differences of Metabolome Profiles

#### Dormancy

**FIGURE 4:**
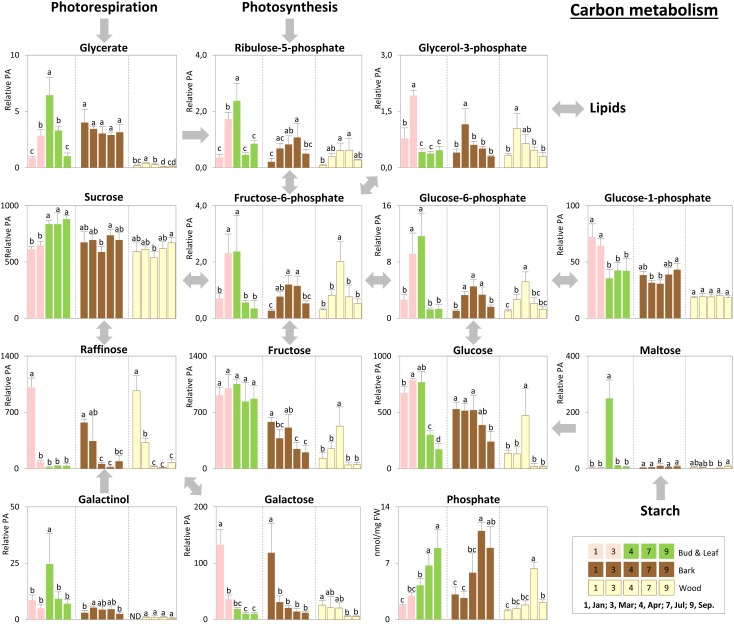
Metabolic changes in carbon metabolism. Changes of the selected metabolites in carbon metabolism in poplar during annual growth. Data represent mean values of five biological replicates in each organ/tissue for each season. Error bars represent SD. Different letters represent statistically significant differences (*P* < 0.05) between seasons in each organ/tissue using Tukey’s test (**Supplementary Table S1**). Relative PA, relative peak area; ND, under detection limit.

**FIGURE 5:**
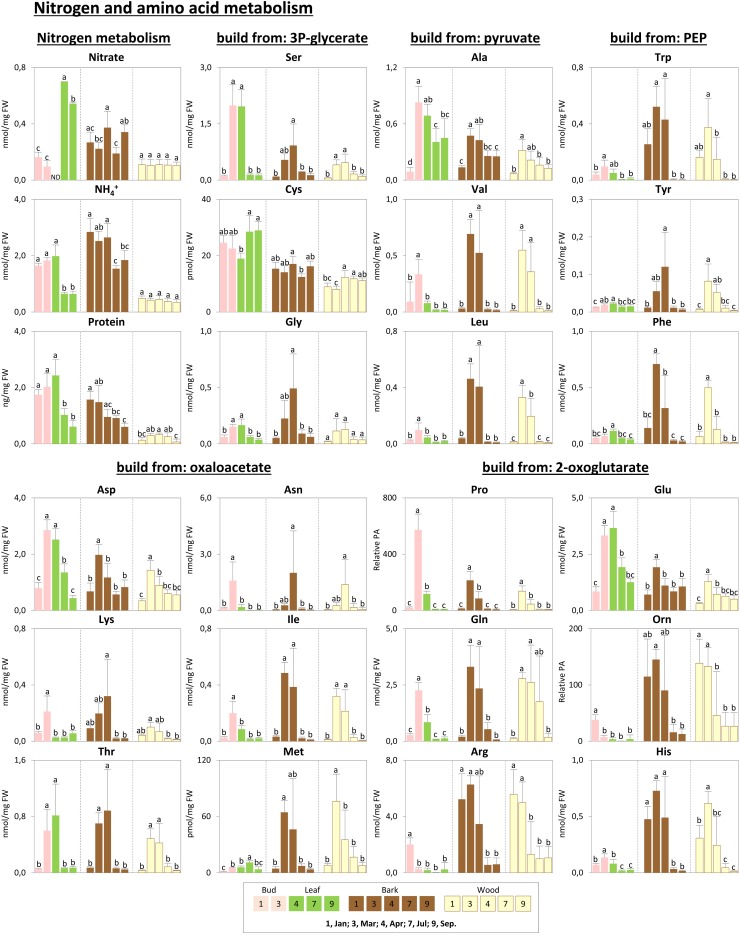
Metabolic changes in nitrogen, protein, and amino acid metabolism. Changes of the selected metabolites in nitrogen, protein, and amino acid metabolism in poplar during annual growth. Data represent mean values of five biological replicates in each organ/tissue for each season. Error bars represent SD. Different letters represent statistically significant differences (*P* < 0.05) between seasons in each organ/tissue using Tukey’s test (**Supplementary Table S1**). Relative PA, relative peak area; 3P-glycerate, 3-phosphate-glycerate; PEP, phosphoenolpyruvate; Orn, ornithine; ND, under detection limit.

**FIGURE 6:**
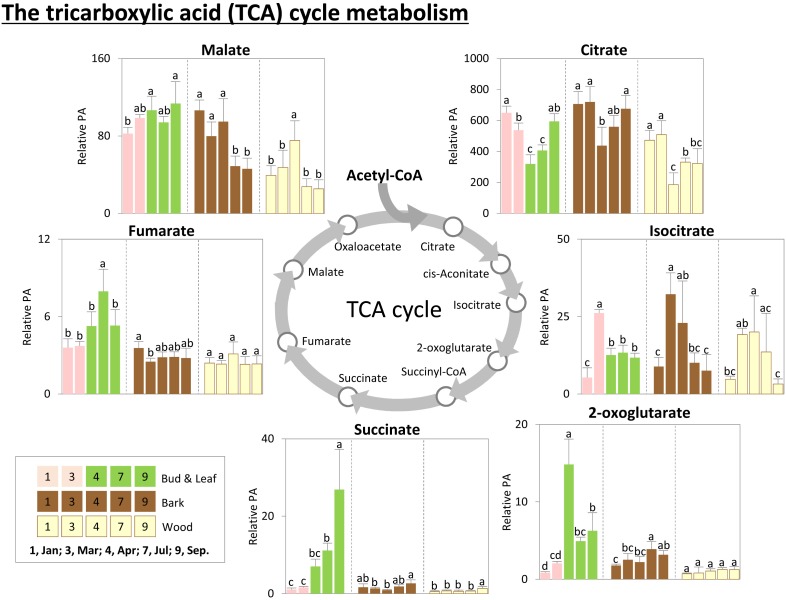
Metabolic changes in the tricarboxylic acid (TCA) cycle metabolism. Changes of the selected metabolites in the TCA cycle metabolism in poplar during annual growth. Data represent mean values of five biological replicates in each organ/tissue for each season. Error bars represent SD. *Different letters* represent statistically significant differences (*P* < 0.05) between seasons in each organ/tissue using Tukey’s test (**Supplementary Table S1**). Relative PA, relative peak area.

**FIGURE 7:**
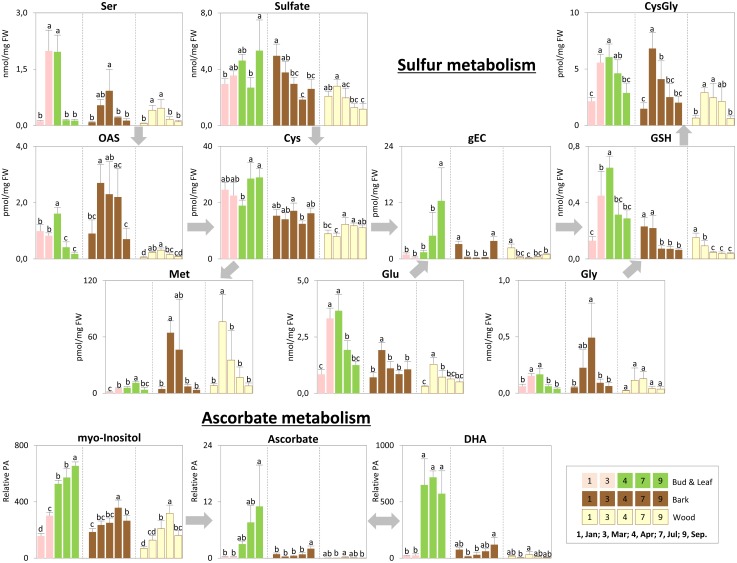
Metabolic changes in sulfur and ascorbate metabolism. Changes of the selected metabolites in sulfur and ascorbate metabolism in poplar during annual growth. Data represent mean values of five biological replicates in each organ/tissue for each season. Error bars represent SD. *Different letters* represent statistically significant differences (*P* < 0.05) between seasons in each organ/tissue using Tukey’s test (**Supplementary Table S2**). Relative PA, relative peak area; OAS, *O*-acetylserine; gEC, gamma-glutamylcysteine; GSH, glutathione; DHA, dehydroascorbate.

Storage compounds such as triacylglycerol (TAG) that indicate lipid storage (Sauter and van Cleve, 1994), Arg and ornithine known as N-storage amino acids (Schneider et al., 1996; Gessler et al., 1998b), as well as the sugars raffinose and galactose (**Figures 3–5**), were highest during dormancy, i.e., in January, in buds/leaves, and twig bark and wood. The N-storage compounds Arg and ornithine (**Figure 5**) remained high in the bark and wood during March prior bud break. In dormant buds, low metabolic activity was indicated by low or lowest levels of a great number of metabolites from groups 3, 4, and 5 (**Figure 3**). For primary metabolism these metabolites included: fumarate, succinate, isocitrate, 2-oxoglutarate (**Figure 6**), sugars such as glycerate, ribulose-5P, fructose-6P, glucose-6P, and myo-inositol (**Figure 4**). In addition, almost all amino acids except Arg (**Figure 5**), plus GSH, γ-glutamylCys (gEC) and CysGly (**Figure 7**) showed lowest levels during winter in January. DHA, ascorbate, salicylate, sinapate, benzoate, which are all known to be involved in defense reactions were found in high abundances in dormant buds and in buds before bud break in March; the level of sulfate, nitrate, and P_i_ showed lowest levels during dormancy and in March prior bud break (**Figures 4**, **5**, **7**; Netzer et al., 2018b). In twig bark and wood, metabolites indicating respiratory activity revealed lowest levels, i.e., isocitrate in group 2, OAS, fructose-6P and glucose-6P in group 3, glycerate and ribulose-5P (**Figures 4**, **6**). Except for Arg that was highest during dormancy, the abundance of almost all free amino acids showed lowest levels in winter during dormancy that were comparable to summer/autumn levels (groups 1, 2, and 3) (**Figure 5**). The observed accumulation of sulfate, the GSH precursor gEC and GSH itself in the bark and during dormancy (**Figure 7**) is consistent to earlier studies with poplar (Dürr et al., 2010) and beech (Herschbach and Rennenberg, 1996) and indicates sulfur storage.

#### Spring

Increasing metabolic activity in spring can be concluded from several primary metabolites, which were all highest in buds/leaves, bark, and wood in March and April. These metabolites include glycerol-3P and isocitrate in group 2, OAS, beta-alanine, fructose-6P and glucose-6P in group 3, glycerate, and ribulose-5P (**Figures 4**, **6**). In addition, after bud break expanding leaves showed peak abundance of maltose, a product of starch degradation (**Figure 4**). This was accompanied by increasing abundances of organic acids of the TCA cycle, i.e., malate, 2-oxoglutarate, and succinate (**Figure 6**). Almost all free amino acids (except Arg) peaked in buds/expanding leaves in spring with a predominant enrichment prior to bud break for Val, Leu, Lys, Asn, Ile, proline, and Gln (**Figure 5**). In the bark and wood, only glycerol-3P but no other sugar (**Figure 4**) peaked in March prior to bud break. This coincides with highest isocitrate levels in both, bark and wood (**Figure 6**), and indicates release of dormancy. Remarkably, during spring before bud break, the lowest levels of DHA and ascorbate of the whole year were observed in the twig organs/tissues, which were studied, namely bud/leaves, bark and wood (group 5) (**Figure 7**). Regarding sulfur metabolism, CysGly a degradation product of GSH (Kumar et al., 2015), strongly increased and showed the highest level in March prior to bud break.

#### Summer

After bud break, metabolites of groups 4 and 5, which even increased in expanding leaves (April) remained high during summer/autumn indicating metabolic activity. Noteworthy compounds include P_i_, myo-inositol, nitrate, fumarate, 2-oxoglutarate, DHA, ascorbate, and succinate. The sugar-Ps fructose-6P, glucose-6P, and ribulose-6P declined in leaves after bud break to the levels still observed in winter during dormancy (**Figure 4**). A similar pattern was found for free amino acids (**Figure 5**), which all declined after bud break (1 April, 2014) to low summer/autumn levels that are also comparable to the levels found in winter buds. Furthermore, changes in sugar abundances that indicate steady state metabolism in summer leaves were observed. Sucrose increased after bud break in leaves whereas glucose and glucose-1P declined and remained at low levels until autumn in September (**Figure 4**). Simultaneously, succinate, fumarate, malate, and 2-oxoglutarate metabolites of the TCA cycle (**Figure 6**), but also DHA and ascorbate (**Figure 7**) started to increase after bud break in the expanding leaves and remained at this level or even further increased during the ongoing vegetative growth period (group 5). In contrast, citrate the first product of the TCA cycle, was at its lowest level after bud break in April, but increased until autumn to the level still observed in winter (**Figure 6**). The anions nitrate and P_i_ showed highest abundances in summer and autumn leaves whereas the level of ammonium was lowest in summer (July) and autumn (September) (**Figures 3**, **5**, **6**; Netzer et al., 2018b).

In twig bark and wood, glucose, fructose, and the organic acids malate and isocitrate continuously declined after bud break, whereas ribulose-5P, fructose-6P, and glucose-6P peaked in April and declined thereafter (**Figure 4**). The level of free amino acids in bark and wood (groups 1, 2, and 3) strongly declined after bud break (**Figure 5**). During summer and autumn, i.e., in July and September, the levels of free amino acids in poplar buds/leaves and twig bark and wood were similar to the levels in winter during dormancy. Most primary metabolites in twig bark and wood showed only minor seasonal changes. These changes included sucrose, glycerate, Cys, 2-oxoglutarate, fumarate, succinate, malate, citrate, and glucose-1P (**Figures 4**, **6**). In addition, ethanolamine, sinapate, benzoate as well as adenine and uracil revealed only minor fluctuations during the annual course (**Figure 3**). However, sulfate was highest during dormancy and spring (**Figure 7**), P_i_ showed its highest level during summer (group 4; Netzer et al., 2018b). The nitrate content did not vary throughout the year, neither in bark nor in wood (**Figure 5**), thus showing a different pattern compared to both, sulfate and P_i_.

#### Autumn

In the leaves the organic acids citrate and succinate increased (**Figure 6**). gEC, the precursor of GSH synthesis (Strohm et al., 1995) reached its highest level (**Figure 7**), while GSH itself declined. In twig bark and wood, raffinose started to increase (**Figure 4**), isocitrate in the wood declined to its winter level and gEC increased in both bark and wood (**Figure 7**).

### Characteristics of Lipidome Profiles by Lipid Classes

**FIGURE 8:**
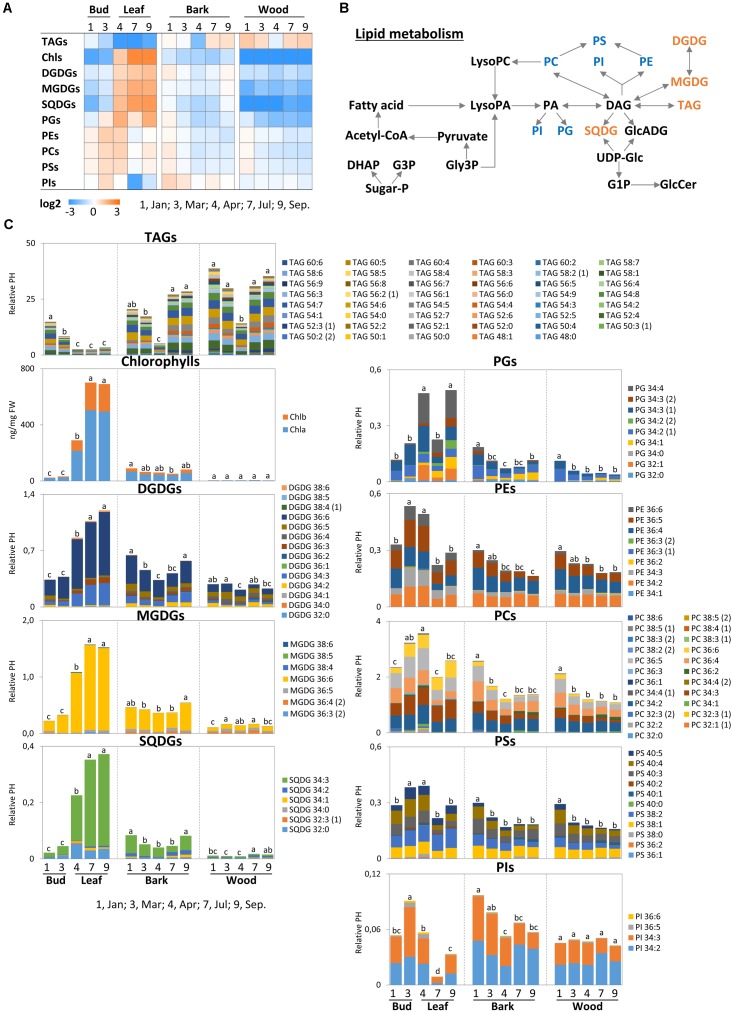
Metabolic changes in lipid metabolism. Changes of the selected metabolites in lipid metabolism in poplar twigs during annual growth. **(A)** Changes of total lipid in each class. **(B)** Lipid metabolic pathway according to Gardocki et al. (2005); Boudière et al. (2014), Guschina et al. (2014); Kobayashi (2016), and Xue et al. (2017). **(C)** Changes of individual lipid molecular species in each class. Log2 ratios of fold changes from the average value of all organs/tissues and seasons are given by shades of *red* or *blue colors* according to the scale bar. Data represent mean values of five biological replicates in each organ/tissue for each seasons. *Different letters* represent statistically significant differences (*P* < 0.05) between seasons in each organ/tissue with the total lipid values in each class using Tukey’s test. Statistical analysis of individual lipid species was performed using Tukey’s test (**Supplementary Table S2**). Relative PH, relative peak height; DHAP, dihydroxyacetonephosphate; Sugar-P, sugar-phosphate; G3P, glucose-3-phosphate; Gly3P, glycerol-3-phosphate; LysoPC, lysophosphatidylcholine; LysoPA, lysophosphatidicacid; GlcADG, glucoronosyldiacylglycerol; UDP-Glc, uridine-diphosphate-glucose; G1P, glucose-1-phosphate; GlcCer, glucosylceramides; TAGs, triacylglycerides; Chls, chlorophylls; MGDGs, monogalactosyldiacylglycerols; DGDGs, digalactosyldiacylglycerols; SQDGs, sulfoquinovosyldiacylglycerols; PGs, phosphatidylglycerols; PEs, phosphatidylethanolamines; PCs, phosphatidylcholines; PSs, phosphatidylserine; PIs, phosphatidylinositols; DAGs, diacylglycerols.

Generally, lipids carry structural functions in membranes or have a storage function and compositions react to environmental cues such temperature and drought to assure optimal membrane fluidity (Oliver et al., 2001; Degenkolbe et al., 2012). The metabolite pattern of lipids is highly complex and varies between tissues and during the annual time course. When focusing on lipid classes, lipid response patterns can be assigned to three different categories, when normalized over all values and tissues (**Figure 8A**). First, TAGs abundance, indicative of oil storage (Hills, 2004), was lowest in leaves during spring and summer. In twig bark and wood, lowest abundance of TAGs was found in April after bud break. Second, the chloroplast lipids MGDG, DGDG and sulfoquinovosyldiacylglycerol (SQDG) grouped together with PG. All these classes displayed low levels in wood and bark across all seasons, but highest levels in leaves during the vegetation period (**Figure 8A**). This pattern exactly follows the accumulation of chlorophyll (**Figure 8A**). Third, the remaining phospholipids, phosphatidylinositols (PIs), phosphatidylethanolamines (PEs), phosphatidylcholines (PCs), and phosphatidylserines (PSs) showed a different pattern to the previous group. The variance of their abundance was less pronounced but highest levels of PE, PC, and PS were observed during spring in buds, while lowest levels were found in the bark and wood during summer and autumn.

### Seasonal Differences in Lipidome Profiles Including Individual Lipids

In buds/leaves the abundance of TAGs that contribute to oil storage in plants (Sauter and van Cleve, 1994; Graham, 2008), was highest in dormant buds and in spring shortly before bud break (**Figure 8C**). However, TAG in buds starts to decline prior to bud break and reached its final low level in April after bud break. A preferential TAG species that contributes to this decline could not be identified (**Supplementary Table S3**). The abundances of the diacylglycerols DGDG, MGDG, SQDG, and the phospholipid PG were low in dormant buds and in March prior to bud break. Their abundances in leaves started to increase after bud break in April and remained high during summer/autumn. Mostly, one or two diacylglycerol species contributed to these changes, i.e., MGDG 36:6, DGDG 34:3 and 36:6, SQDG 32:0 and 34:3 (**Figure 8C** and **Supplementary Tables S2**, **S3**). Except for PG, the phospholipids (PC, PE, and PS) were highest in expanding leaves during spring, i.e., March and April, but similarly abundant during dormancy, summer and autumn. Only PI peaked in March prior bud break, declined in April and showed the lowest level in summer. These changes could not be attributed to a particular phospholipid species, because the abundance of all phospholipids changed in a comparable manner.

The lipidome profile of both twig tissues, bark and wood, showed minor changes during annual growth with one exception (**Figure 8C**). TAG abundance was lowest in April after bud break indicating oil mobilization. All TAG species contributed equally to the decline in TAG in both, bark and wood. In the bark, DGDG and SQDG showed lowest levels in April after bud break. DGDG, SQDG, and TAG were highest during autumn (September). This seems surprising because MGDGs, DGDGs, and SQDG are chloroplast lipids (Watanabe et al., 2013; Nakamura, 2017). Bark photosynthesis (Aschan and Pfanz, 2003; Wittmann and Pfanz, 2007) and the formation of amyloplasts build for starch storage in the wood and bark (Sauter et al., 1996) are responsible for the occurrence of these lipids. MGDG levels in the bark, however, remained unaffected throughout the entire annual growth (**Figure 8C**), as also observed for MGDG, DGDG, and SQDG in the wood. The abundance of all phospholipids (PG, PE, PC, PS, and PI in the bark only) was highest during dormancy, but similar during the other seasons. PI was dominated by two species, namely PI 34:2 and 34:3 (**Figure 8C** and **Supplementary Tables S2**, **S3**).

## Discussion

**FIGURE 9:**
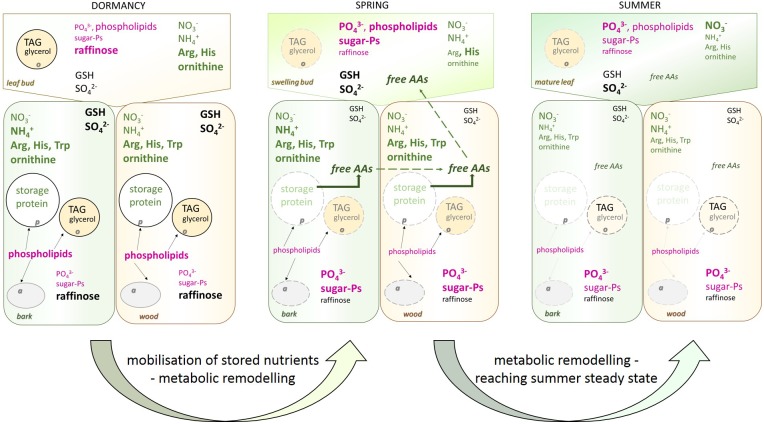
Overview of seasonal changes in metabolite and lipid abundances in poplar twigs. Seasonal differences in metabolite and lipid abundances in the twig buds/leaves, bark, and wood is summarized for dormancy, spring and summer. *Bold and bigger font* demonstrated higher abundance of the compound in relation to other season(s). Vice versa *smaller letter* indicate lower amount of the compound in relation to other season(s). *Magenta* written compounds relate to the P metabolism, *green* written compounds relate to the N nutrition while *black* or *gray* written compounds refer to the C and S metabolism. *Light gray* and *light green* fonts indicated the low abundance of TAG, glycerol, and of protein at this time of the year. *Black arrows* show the places of phospholipids. *Broken lines* of protein body, amyloplasts and oleosome in spring indicated their degradation. a, amyloplast; o, oleosome; p, proteine body; AAs, proteinogenic amino acids.

The present study on the metabolome and lipidome of poplar twigs during annual growth highlights metabolite changes due to C, N, and S storage and mobilization, supports the provision of P metabolite changes due to their demand and the lack of any P storage in poplar (**Figure 9**). During dormancy, phospholipids in the bark and wood were highest, probably due to the support of frost-hardening (Yoshida and Sakai, 1973; Degenkolbe et al., 2012) and most reasonable to their requirement for extraplastidic membranes such as amyloplasts, oleosomes, and protein bodies. Consistently, sugar-Ps increased when phospholipids declined and metabolic activity in spring increased in poplar plants at the beginning of vegetative growth. Furthermore, the accumulation of phospholipids and sugar-Ps during leaf development arises from *de novo* synthesis and the P_i_ uptake by the roots. Hence, the metabolome and lipidome profiles of the present study provide a comprehensive view on (i) storage and mobilization of C, N, and S compounds in poplar twig organs/tissues during annual growth and (ii) changes in metabolic activity during annual growth. In addition, they (iii) verify the absence of any P storage, and (iv) show that the composition of P_org_ still is subjected to strong seasonal variations due to changed P_org_ requirements and metabolic activities.

### Accumulation of Phospholipids in Bark and Wood During Dormancy Relates to Their Need for Extraplastidic Membranes

Phospholipids (PS, PG, PE, and PC) peaked during dormancy in poplar bark and wood when the level of P_org_ was lowest. Hence, it is unlikely that phospholipids accumulated due to P storage. Instead, phospholipid accumulation in poplar twigs can be associated with frost hardening (Yoshida and Sakai, 1973; Latsague et al., 1992; Moellering et al., 2010). Moreover, it has to be taken into account that PC and PE are major extraplastidic lipids (Nakamura, 2017). During dormancy, a large number of protein bodies, oleosomes, and amyloplasts are established in twig tissues of poplar for protein, oil, and starch storage, respectively (Sauter and Kloth, 1987; Sauter and van Cleve, 1994; Sauter et al., 1996; Sauter and Wellenkamp, 1998). Hence, instead of phospholipid storage as found for beech (Netzer et al., 2018a), the need of phospholipids in extraplastidic membranes may explain their high abundance during dormancy. After bud break, both, the decline of phospholipids and the increase of sugar-Ps in bark and wood coincide with mobilization of storage compounds such as proteins and the storage lipids TAG, all diminishing after bud break. Hence, protein bodies that store protein and oil storing oleosomes become degraded possibly by autophagy (Zientara-Rytter and Sirko, 2016) and metabolic remodeling caused the release of membrane lipids (Sauter and Kloth, 1987; Sauter and van Cleve, 1994; Sauter et al., 1996; Sauter and Wellenkamp, 1998; **Figure 9**). The increasing abundances of sugar-Ps in bark and wood fits well to this assumption and could be an indication of (i) increasing metabolic activity, (ii) synthesis of P_org_ for its transport into developing buds as observed for beech (Netzer et al., 2018a), or (iii) gluconeogenesis from fatty acids catabolism after cleavage from storage lipids (Graham, 2008). Transport of sugar-Ps to young developing poplar leaves is indicated by highest abundance of P_org_ in leaves after bud break. However, against this assumption, P_tot_ in the bark and wood increased instead decreasing and P_i_ uptake seemed to fulfill the P demand of growing tissues of the poplar leaves in spring (Netzer et al., 2018b). This clearly supports the assumption that the decline of phospholipids in poplar twig bark and wood coincides with membrane degradation and is not a feature of P mobilization from storage pools as observed for beech (Netzer et al., 2018a).

In leaf buds before and after bud break, all phospholipids (PE, PC, PS, and PI) were higher when compared to winter buds and summer leaves. After bud break, the level of phospholipids declined when simultaneously DGAG, MGDG, and SQDG levels increased. These glycolipids are thylakoid membranes constituents (Watanabe et al., 2013; Boudière et al., 2014; Siebers et al., 2015; Nakamura, 2017) and their increase coincides with increasing chlorophyll levels at the start of the vegetative growth. TAG breakdown in poplar leaves during spring may provide the fatty acids required for phospholipid synthesis (**Figure 8B**). At the same time, peak amounts of sugar-Ps (ribulose-5P, glucose-6P, and fructose-6P) were observed in both, buds before bud break and in expanding/developing leaves after bud break (**Figure 4**). Considering that in developing buds/leaves highest levels of P_org_ were detected and that the P_i_ uptake in spring fulfills their P demand (Netzer et al., 2018b), it can be concluded that P_i_ taken up by the roots is immediately used for the synthesis of phospholipids and sugar-Ps. In conclusion, differences in demand during the annual growth of poplar leaves, bark and wood, but not its function in P storage determines the P_org_ abundance in buds/leaves, as well as in bark and wood of poplar twigs.

### Carbon Metabolism of Poplar Twigs Shows Oil Storage and Mobilization As Well As Increasing Metabolic Activity in Spring

Carbon storage compounds such as glycerol, galactose, and raffinose were highest during dormancy in twig bark and wood as previously observed (Sauter and van Cleve, 1994; Sauter and Wellenkamp, 1998). The present study shows that these storage compounds are complemented by TAG accumulation in dormant buds (**Figures 3**, **4**, **8**). Besides starch, storage lipids in the form of TAGs are well-known C storage compounds in numerous plants (Athenstaedt and Daum, 2006; Chapman et al., 2012) and might contribute to frost hardening (Degenkolbe et al., 2012). In the present study, TAG accumulation in dormant buds and its decline during bud break in spring indicate the use of this C source for leaf expansion and growth (**Figures 8C**, **9**) and fits well with the peak abundance of glycerol-3P in March prior bud break (**Figure 4**). Glycerol-3P and fatty acids produced from TAG degradation can be channeled into glycolysis and further on into the TCA cycle for respiration or can be used via gluconeogenesis for the synthesis of structural carbohydrates via glucose-6P and fructose-6P (Graham, 2008). This may be of high importance during bud break, when the need for structural carbohydrates is high. The high abundance of sugar-Ps (fructose-6P and glucose-6P) in poplar twigs in spring and early summer indicates high metabolic activity (**Figure 4**) that is furthermore evident from highest free amino acid abundances, but also from highest levels of isocitrate in all twig tissues as well as of 2-oxoglutarate and ribulose-5P in leaves in spring (**Figures 4**, **6**).

### Changes of the Nitrogen Metabolome During Annual Growth Showed Nitrogen Storage and Mobilization

Protein storage in the bark (**Figures 5**, **9**; Coleman et al., 1991; Clausen and Apel, 1991; Millard and Grelet, 2010; Wildhagen et al., 2010) was complemented by protein accumulation in dormant leaf buds (**Figure 5**). Further N-storage compounds identified in the present study include Arg and ornithine, as well as His and Tyr that were highly abundant in twig bark and wood during dormancy. In previous studies, high levels of Arg have been described in leaves of beech trees and in below ground tissues of Norway spruce from a field site (Höglwald, Germany) exposed to high loads of ammonium and nitrate (Schneider et al., 1996; Gessler et al., 1998b), in poplar wood during spring (Larisch et al., 2012), and in poplar bark during dormancy (Wildhagen et al., 2010). The xylem sap of evergreen *Quercus ilex* exhibited high levels of ornithine from January until April supposed to originate from Arg degradation after protein break down (Nabais et al., 2005). All these data from studies with different tree species support the conclusion of additional N-storage in form of amino acids in poplar.

In spring, almost all free amino acids reached highest levels before and after bud break in twig bark and wood (Asn, Gln, Gly, His, Ile, Leu, Lys, Met, Pro, Phe, Ser, Thr, Trp, Tyr, and Val), indicating storage protein degradation. This view is supported by declining protein levels in the bark but not in the wood (**Figure 5**). Protein storage in the bark was detected first in the 1980s (Sauter and Wellenkamp, 1988; Sauter et al., 1989; Wetzel et al., 1989) and different storage protein families were classified in trees (Coleman et al., 1991; Cooke and Weih, 2005; Wildhagen et al., 2010). Bark storage protein accumulates in protein bodies surrounded by membranes consisting of phospholipids (Nakamura, 2017). Protein break down in the bark from protein bodies is accompanied by a decline in all phospholipids that supports the assumption of protein body degradation in the present study.

Although free amino acids peaked in twig wood during spring, the protein level was highest in spring and early summer (**Figures 5**, **9**). Hence, the higher amino acid abundance in the wood of poplar twigs at this time of the year could hardly be explained by protein degradation in the wood itself. During bud break the abundance of free amino acids in the xylem sap strongly increased in deciduous and evergreen trees (Schneider et al., 1994; Millard et al., 1998; Nabais et al., 2005; Millard and Grelet, 2010; Netzer et al., 2018a). This may result in increasing amino acid levels of the entire wood as analyzed in the present study and, thus, may explain the high abundance of free amino acids in the wood despite increasing protein levels in spring. Furthermore, phloem-to-xylem exchange of amino acids as observed for beech (Gessler et al., 2003) could induce amino acid accumulation in the wood after protein degradation in the bark. Moreover, degradation of stored proteins in roots, what to the authors’ knowledge has not been shown so far, can be another source for the amino acid accumulation in poplar twig wood due to its allocation in the xylem. On the other hand, increasing protein levels in the wood during spring are not surprising, since increasing metabolic activity can be expected at this time of the year (Okumotoa and Pilot, 2011; Tegeder, 2012) and is evident from enhanced sugar and sugar-Ps contents (fructose, glucose, fructose-6P, and glucose-6P), from higher levels of malate and isocitrate, and from P_i_ accumulation (**Figures 4**, **6**).

The N supply to developing and expanding poplar leaves in spring via bark storage protein degradation and subsequent xylem transport seems to be supplemented by protein storage in dormant buds itself. Highest protein levels in leaf buds were found during dormancy but also in spring after leaf expansion in April (**Figure 5**). Whether the proteins in dormant poplar leaf buds belong to the vegetative storage proteins or to functional protein classes needs to be investigated in further studies. In addition, newly synthesized proteins due to increasing metabolic activity in expanding leaves can supplement the protein pool of dormant leaf buds.

When the leaves are fully expanded in July but also in autumn, the protein level amounted to only 1/3 compared to dormant and spring buds. Simultaneously, nitrate in mature leaves was nearly sixfold higher, whereas NH_4_^+^ declined to one third compared to dormant buds. This might be an indication either of a surplus of nitrate uptake and allocation to the leaves via xylem transport followed by nitrate storage in vacuoles and/or of downregulated nitrate reduction and assimilation in mature leaves in summer/autumn. Consistently, nitrate reductase activity in poplar twigs and/or roots was lower compared to leaves, and highest leaf nitrate reductase activity was observed in young leaves (Black et al., 2002). High levels of allantoin during the whole vegetation period from bud break until late summer also indicate a surplus of nitrogen in mature poplar leaves. Allantoin is a product of urate turnover that is produced from nucleotide (AMP and GMP) degradation (Nikiforova et al., 2005; Watanabe et al., 2014). In plants, the main function of purine catabolism is recycling nitrogen for its reuse to promote new growth and reproduction (Zrenner et al., 2006). Therefore, accumulation of allantoin in mature poplar leaves of the present study (**Supplementary Table S1**) does not seem to be used in recycling N, but rather indicates sufficient N availability in the soil that is also evident from Ala and Glu accumulation (**Figure 5**).

### Metabolic Remodeling Showed S Storage and Mobilization

In the present study storage of sulfur occurred as sulfate in poplar twig bark and wood (**Figures 7**, **9**; Herschbach and Rennenberg, 1996; Dürr et al., 2010; Malcheska et al., 2013), but in contrast to beech (Herschbach and Rennenberg, 1996) not in dormant poplar buds. Another well-known sulfur compound involved in S storage is GSH. GSH was highest during dormancy in bark and wood, but not in dormant leaf buds. Hence, different to beech, sulfur storage in buds seems not to be relevant for poplar (Herschbach and Rennenberg, 1996). Sufficient sulfur may become available in developing buds from storage protein degradation in the bark and subsequent sulfate and/or GSH transport in the xylem to the developing buds (**Figure 7**; Dürr et al., 2010; Malcheska et al., 2013).

In leaves, GSH increased during spring, when the S storage pool of the twig was mobilized, indicated by a decline of GSH in bark and wood (**Figure 7**). Transport of GSH and/or Cys in the xylem has been described for several deciduous tree species. Depending on the tree species, GSH or Cys (Cys in beech: Schupp et al., 1991; Rennenberg et al., 1994; GSH in poplar: Schneider et al., 1994, and in *Abies*: Escher et al., 2003) as well as sulfate were enriched in the xylem sap in spring. In this context, highest CysGly levels in the bark but also in leaves and wood in spring are noteworthy (**Figure 7**). CysGly is a degradation product from GSH (Kumar et al., 2015). Thus, its high level in poplar bark and buds indicate enhanced GSH degradation in spring, especially in leaves which are sinks for sulfur at this time of the year (Dürr et al., 2010) when synthesis of functional proteins for leaf development requires a high availability of reduced sulfur. Hence, the high GSH level in leaves originating from xylem transport and the high level of CysGly indicate GSH degradation to provide Cys for functional protein synthesis.

## Conclusion

The metabolite and lipid profile of poplar twig organs/tissues showed seasonal and tissue specific differences that can be linked to C, N, and S storage and mobilization processes and support the lack on any P storage. TAG break down in twig bark and wood and in buds indicates C mobilization to support growth and development of leaves in spring. Fatty acids from TAG degradation in poplar leaves in spring may be used as C source for phospholipid and chlorophyll synthesis, but also for the formation of structural carbohydrates via gluconeogenesis. Accumulation of phospholipids during dormancy in bark and wood seems to be due to its need in membranes of amyloplasts, oleosomes and protein bodies, i.e., in subcellular compartments with high abundance during dormancy. Protein accumulation as N storage pool in buds and bark is complemented by the accumulation of the N rich amino acids Arg, Orn, and His that are mobilized in spring to support leaf development. Furthermore, sulfur is stored as sulfate and GSH in bark and wood. Thus, the present results demonstrated C, N, and S storage and mobilization that is not observed for P. Nevertheless, remodeling of organic-P compounds revealed their distinct needs in specific processes depending on the season. This nutritional strategy for P differs from the climax tree species *Fagus sylvatica* and can be linked to the growth behavior and growth habitat of *P.* x *canescens* in nutrient rich floodplains.

## Author Contributions

MW performed the metabolome analyses, did all data analyses, and created the figures. TT and AF performed GC-TOF/MS analysis for primary metabolites. IO and YB performed LC/ESI-MS analysis for lipids. FN performed harvest of poplar samples and contributed to data interpretation. DD performed and evaluated IRMS measurements. HR contributed to designing the research question and experimental approach. RH supervised metabolome profiling and data calculation. CH designed the research question, provided suggestions on figure illustration, and wrote the manuscript. All authors discussed the results and commented on the manuscript.

## Conflict of Interest Statement

The authors declare that the research was conducted in the absence of any commercial or financial relationships that could be construed as a potential conflict of interest.

## Supplementary Material

TABLE S1 Mean values (±SD) of all metabolites and results from Tukey’s test for each tissue.

TABLE S2 Mean values (±SD) of all lipids and results from Tukey’s test for each tissue.

TABLE S3 Heat map of lipid changes in each tissue of poplar during annual growth.
